# A STAT1-Knockout Mouse Model for Chapare Virus Infection and Pathogenesis

**DOI:** 10.3390/v18030388

**Published:** 2026-03-20

**Authors:** Stephanie R. Monticelli, Ana I. Kuehne, Thomas G. Batchelor, Joshua B. Richardson, Zebulon Lapoint, Jennifer L. Williams, Susan R. Coyne, Jo Lynne W. Raymond, Xiankun Zeng, Christopher P. Stefan, Jeffrey W. Koehler, Jeffrey R. Kugelman, Andrew S. Herbert

**Affiliations:** 1 Viral Immunology Branch, Virology Division, United States Army Medical Research Institute of Infectious Disease, Ft. Detrick, Frederick, MD 21702, USA; ana.i.kuehne.civ@health.mil (A.I.K.); thbatche@ucsc.edu (T.G.B.); andrew.s.herbert4.civ@health.mil (A.S.H.); 2 Henry M. Jackson Foundation for the Advancement of Military Medicine, Bethesda, MD 20817, USA; 3 Oak Ridge Institute for Science and Education, Oak Ridge, TN 37830, USA; 4 Center for Genome Science, United States Army Medical Research Institute of Infectious Disease, Ft. Detrick, Frederick, MD 21702, USA; joshua.b.richardson2.civ@health.mil (J.B.R.); zebulon.r.lapoint.ctr@health.mil (Z.L.); jennifer.l.williams410.ctr@health.mil (J.L.W.); jeffrey.r.kugelman.mil@health.mil (J.R.K.); 5 Cherokee Nation Integrated Health, Catoosa, OK 74015, USA; 6 Developmental Diagnostics Branch, Diagnostic Systems Division, United States Army Medical Research Institute of Infectious Disease, Ft. Detrick, Frederick, MD 21702, USA; susan.r.coyne.civ@health.mil (S.R.C.); christopher.p.stefan.civ@health.mil (C.P.S.); 7 Pathology Division, United States Army Medical Research Institute of Infectious Disease, Ft. Detrick, Frederick, MD 21702, USA; jolynne.w.raymond.civ@health.mil (J.L.W.R.); xiankun.zeng.civ@health.mil (X.Z.); 8 Operational Diagnostics Branch, Diagnostic Systems Division, United States Army Medical Research Institute of Infectious Disease, Ft. Detrick, Frederick, MD 21702, USA; jeffrey.w.koehler4.civ@health.mil

**Keywords:** Chapare virus, STAT1 knockout, mouse model, arenavirus, Chapare hemorrhagic fever

## Abstract

Chapare virus (CHAPV) is an *Arenaviridae* family member and causative agent of Chapare hemorrhagic fever (CHHF). Endemic to Bolivia, CHAPV was found to be the cause of several outbreaks of CHHF in Bolivia in 2003 and 2019 with high case-fatality rates and instances of human-to-human transmission. The pathogenesis of CHAPV infection is poorly understood, and no vaccines or antivirals are available, in part due to a dearth of available animal models. Mice lacking signal transducer and activator of transcription 1 (STAT1^-/-^) have been shown to succumb to infection by related arenaviruses, including Machupo virus, and were investigated for their susceptibility to CHAPV infection. Challenge with CHAPV resulted in partial lethality in STAT1^-/-^ mice with a biphasic disease course characterized by initial viral load and pathology in the spleen and liver followed by inflammation and high viral titers in the brain and spinal cord that immediately preceded mortality. Adaptation in the brains of STAT1^-/-^ mice resulted in a fully lethal mouse-adapted CHAPV variant, with a similar biphasic disease course, but virus in tissues was detected more proximal to challenge. The result of this study is a lethal small-animal rodent model for CHAPV that recapitulates many aspects of human CHAPV disease.

## 1. Introduction

Chapare mammarenavirus (CHAPV) is an arenavirus endemic to Bolivia that causes severe hemorrhagic fever in humans [1]. The first recorded outbreak occurred in 2003 within the Cochabamba Department of Central Bolivia [1]. Several cases of viral hemorrhagic fever were reported, and one death occurred resulting in the initial isolation and discovery of the novel arenavirus. A second outbreak occurred more recently in 2019 with symptoms presenting in nine individuals resulting in the death of four [2,3]. Evidence suggests that three of these cases were spread through nosocomial transmission [2,3]. Continued sporadic cases, as recent as January 2025, continue to be reported. Agricultural workers make up a large proportion of individuals infected with CHAPV. This finding coincides with that of other arenaviruses, with rodents being the primary vector of transmission, and suggesting that initial infection occurs through direct contact with urine, saliva, and other excretions from a rodent harboring CHAPV [2,3]. Subsequent ecologic investigations to identify a rodent vector found that small-eared pygmy rice rats (*Oligoryzomys microtis*) were positive for CHAPV RNA [2,3]; however, due to the limited amount of data, the natural reservoir of CHAPV is still unconfirmed. In recent years, a sharp increase in viral hemorrhagic fever cases coincides with increased farming and logging in South America [4,5]. Continued urban and agricultural expansion is likely to lead to more frequent interactions between humans and infected rodents, contributing to increased outbreaks of these viral hemorrhagic fevers.

Human CHAPV disease, referred to as Chapare hemorrhagic fever (CHHF), presents as an acute febrile illness with symptoms including nausea, myalgia, vomiting, headaches, mucosal hemorrhaging, and neurological abnormalities [1,2]. In the limited number of recorded cases, death occurred between 9 and 21 days following the onset of symptoms. Neurological symptoms occurred in 5 out of 9 patients in the 2019 outbreak and included paraparesis, disorientation, seizures, and agitation [2]. CHHF disease presentation has many commonalities with other new world arenaviruses as well as other infectious agents, such as dengue virus, thus presenting a challenge in the identification and subsequent treatment of CHAPV infection. There currently exists a significant gap of knowledge pertaining to CHAPV including the mode of transmission, natural reservoir, diagnostic testing, and potential for therapeutic interventions. No licensed therapeutics or vaccines currently exist to prevent or treat CHAPV infection. The off-label use of FDA-approved drugs, such as Ribavirin and Favipiravir, have shown some clinical success against other new world arenaviruses, but often must be given at early stages of infection when patients present with non-specific symptoms [6,7], and efficacy has yet to be shown against CHAPV. The most recent outbreaks prove that the virus is still present in nature, lethal to humans, and likely to persist through future outbreaks.

Filling in these gaps and collecting clinically relevant data start with the development of an effective animal model. Until recently, there were no published animal models for CHAPV. The exception is a report showing that Strain 13 guinea pigs are susceptible to CHAPV, succumbing to infection between 14 and 16 days post-infection [8], and a cynomolgus macaque model that recapitulates several key features of human CCHF [9]. However, Strain 13 guinea pigs are not commercially available, limiting their widespread use, and numerous limitations exist in the use of the cynomolgus macaque model, including the fact that it is a partially lethal model, and that non-human primate studies are associated with numerous logistical and financial constraints in addition to availability and ethical considerations. The disease in Strain 13 guinea pigs also lacks some of the typical presentations observed in humans, including neurological manifestations. Several animal models exist for Machupo virus (MACV), a new world arenavirus closely related to CHAPV. Established MACV-specific animal models include non-human primate (NHP), Hartley guinea pig, and several immunodeficient mouse models [10,11,12,13,14,15,16,17]. Specifically, mice lacking signal transducer and activator of transcription 1 (STAT1^-/-^) are susceptible to MACV infection [12,18]. This research extends the use of this immunodeficient mouse model to CHAPV and describes the development of the first lethal small-animal mouse model for Chapare virus. This model recapitulates certain aspects of the major pathological symptoms observed during human disease, including neurological afflictions. The intent of this model is to provide a platform allowing for a greater understanding of the virus and how it may be treated and provide a tool for antiviral therapeutic discovery and evaluation.

## 2. Materials and Methods

### 2.1. Cell Lines

VeroE6 (RRID: CVCL-0574) were obtained from the American Type Culture Collection (ATCC). Cells were cultured in Dulbecco’s Modified Eagle Medium (DMEM) (ThermoFisher Scientific, Waltham, MA, USA) containing glutamine, 10% heat-inactivated (Δ) fetal bovine serum (ΔFBS; Gibco, New York, NY, USA) and 1% penicillin–streptomycin (ThermoFisher Scientific, Waltham, MA, USA). All in vitro infections were conducted using DMEM with glutamine, 2% ΔFBS, and 1% penicillin–streptomycin. Cells were maintained in a 37 °C incubator supplied with 5% CO_2_. Cell lines were not authenticated following purchase.

### 2.2. Virus Stocks

The Chapare virus strain 810419 (GenBank IDs listed in Table 1) was utilized in these studies. This strain was isolated from a fatal case of hemorrhagic fever in the Chapare region of Bolivia in late 2003/early 2004 [1] and subsequently passaged one time in VeroE6 times to obtain a viral stock. The starting stock was sequenced to verify its identity. All virus stocks were grown on VeroE6 cells. VeroE6 cells were inoculated with 0.01 multiplicity of infection (MOI) of CHAPV and incubated for 1 h at 37 °C. Following incubation, fresh media was added to monolayers and incubated for 6 days at 37 °C. Following infection, supernatant was collected, clarified by centrifugation at 4000× *g* for 10 min, and aliquoted following plaque assay to calculate viral titer (4.56 × 10^6^ pfu/mL).

### 2.3. Animal Models

Four–twelve-week-old male and female B6.129S(Cg)-*Stat1*^tm1Dlv^/J mice (STAT1^-/-^; strain #012606; The Jackson Laboratory, Bar Harbor, ME, USA), ranging in weight from 15 to 30 g, were used in all animal challenge experiments. These animals had previously never undergone experimentation and were confirmed to be free of contaminating bacterial or viral pathogens by the vendor. Animals were randomly allocated to experimental groups and provided with food and water ad libitum and housed in individually ventilated cages in groups of 2–10 mice per cage under specific pathogen-free conditions at USAMRIID. Murine challenge studies were conducted under an Institutional Animal Care and Use Committee-approved protocol in compliance with the Animal Welfare Act, Public Health Service Policy, and other federal statutes and regulations relating to animals and experiments involving animals. The facility where this research was conducted (USAMRIID) is accredited by AAALAC International and adheres to principles stated in the Guide for the Care and Use of Laboratory Animals, National Research Council 2011. Mice determined to be moribund (e.g., immobility, lateral recumbency, non-responsiveness to stimulation, inability to eat or drink), in accordance with the USAMRIID IACUC-approved criteria, were promptly euthanized.

### 2.4. In Vivo Challenges

For all studies, STAT1^-/-^ mice were exposed through the intraperitoneal (IP) route to a target dose of 1000 pfu of CHAPV, and these mice were monitored daily for clinical signs of disease, weight loss, and mortality. Challenge dose was confirmed by back-titration using a plaque assay. Mice were scored on a 4-point grading scale, where 1 was defined by decreased grooming and/or ruffled fur, 2 defined by subdued behavior when unstimulated, 3 defined by lethargy, hunched posture and/or subdued behavior even when stimulated, and 4 defined by bleeding, unresponsiveness, severe weakness, or inability to walk. Mice scoring a 4 were considered moribund and were euthanized. Neurological manifestations including circling, ataxia, head tilt, and partial or full hind limb paralysis were considered in the scoring criteria pertaining to how they impacted overall behavior as described above. Mice with hind limb paralysis that could not access food or water were considered moribund. For serial sampling studies, at the indicated days post-challenge (0, 2, 4, 6, 8, 10, 12, 14, 16, 18, 20, 23, 28, and 35 and 4, 8, 12, 18, 20, 23, 29, and 36 days post-challenge for wild-type and mouse-adapted CHAPV, respectively), mice (*n* = 2–5) were euthanized and whole blood, the liver, the spleen, the kidneys, the lungs, the brain, and the spinal cord were collected. Mice were sampled at random. For the wild-type CHAPV serial sacrifice study, 72 mice were challenged and *n* = 5 mice harvested at each time point (only 3 mice sampled on day 0). Mice were separated into four pans of females and four pans of males (maximum of *n* = 10 mice per pan), group weighed by pan, and pan weights reported. For the ma-CHAPV serial sacrifice study, 32 mice were challenged and *n* = 4 mice harvested at each time point (only 1 and 2 mice sampled on days 0 and 36, respectively). Mice were separated into two pans of females (*n* = 8, 10 mice per pan) and two pans of males (*n* = 10, 4 mice per pan), group weighed, and pan weights reported. For both studies, mice that were found dead were not sampled.

### 2.5. Generation of Mouse-Adapted CHAPV

For each passage, STAT1^-/-^ mice were exposed through the IP route to 1000 pfu of challenge stock, and a subset (*n* = 2–5) were harvested for brain tissue at times when they were displaying severe weight loss (>20% weight loss) and clinical signs of disease (clinical score ≥ 3)—between 27 and 38 days post-challenge. The remaining mice (*n* = 5–8) were monitored for lethality for 42 days post-challenge. Tissues were homogenized, and the viral titer was assessed by plaque assay. Tissues from *n* = 1–2 mice with the highest viral load were combined, and 1000 pfu of homogenate was injected via the IP route into naïve STAT1^-/-^ mice for the next passage. In total, 5 passages were completed. Following confirmation of lethality, cell culture stocks of in vivo passaged virus were generated as described above.

### 2.6. Plaque Assay

Whole blood was centrifuged at 12,000× *g* for 10 min to collect serum. Tissues were homogenized in infection media in gentleMACS M tubes (Miltenyi Biotec, Gaithersburg, MD, USA) to generate a 10% tissue homogenate and clarified to remove cellular debris by centrifugation at 4000× *g* for 10 min. Serial dilutions of serum and tissue homogenates were prepared in infection media. Following dilutions, 200 µL from each dilution was inoculated onto VeroE6 cell monolayers in 6-well plates. After adsorption for 1 h at 37 °C, cell monolayers were overlaid with a mixture of 1 part 1% agarose (Seakem ME, Lonza Inc, Rockland, ME, USA) and 1 part 2x Eagle basal medium, 30 mM HEPES buffer, and 2% ΔFBS. Plates were incubated for 8 days at 37 °C, at which point a second overlay supplemented with 5% neutral red was added. Plaques formed by infectious CHAPV were counted 24 h later. Titers are shown as plaque-forming units (PFU)/mL.

### 2.7. ELISA

High-binding half-area plates (Greiner Bio-One, Monroe, NC, USA) were coated with 1:1000 dilution of irradiated Chapare virus (BEI Resources, Manassas, VA, USA; NR-37377) at 25 µL/well and incubated overnight at 4 °C. Approximately eighteen hours later, plates were blocked with blocking buffer (5% milk in 1× phosphate-buffered saline [PBS] with 0.05% Tween-20; PBST) for two hours at ambient temperature. Serum was diluted 1:100 in blocking buffer followed by 2-fold dilutions (final dilution of 1:6400). Following blocking, liquid was removed by flicking, and each dilution was added to plates in duplicate and incubated for two hours at ambient temperature. Plates were washed 3× with PBST and then incubated with either horseradish peroxidase (HRP)-conjugated goat anti-mouse IgG (Jackson ImmunoResearch Laboratories, West Grove, PA, USA) or HRP-conjugated goat anti-mouse IgM (Jackson ImmunoResearch Laboratories, West Grove, PA, USA) for one hour at ambient temperature. Plates were washed 3× with PBST and incubated with the substrate 3,3′,5,5″-Tetramethylbenzidine (TMB) (ThermoFisher Scientific, Waltham, MA, USA) for 30 min at ambient temperature. Plates were read at 450 nm (SpectroMax M5; Molecular Devices, San Jose, CA, USA) after the addition of stop solution (0.16M sulfuric acid). A cutoff value to determine positive signal was calculated as 2.5 standard deviations above the mean optical density (OD) value of naïve, unchallenged sera. A sigmoidal, 4PL curve was fit to interpolate the data and calculated area under the curve (AUC).

### 2.8. Neutralization Assay

Sera samples were heat inactivated at 56 °C for 30 min. CHAPV was incubated with serial 3-fold dilutions of heat-inactivated sera (starting at a dilution of 1:5) for 1 h at 37 °C. The serum–virus mixture was added to monolayers of VeroE6 cells in a 96-well plate at a final multiplicity of infection of 0.15 and incubated for 1 h at 37 °C. The infection medium was removed, and a fresh cell culture medium without serum was added. Then, 48 h post-infection, the culture medium was removed, and plates were fixed in 10% neutral buffered formalin (Valtech, Pottstown, PA, USA) for at least 24 h at 4 °C. The plates were removed from formalin and permeabilized with 0.2% Triton-X for 10 min at room temperature and treated with blocking buffer (5% milk). Infected cells were detected by sequential incubation with anti-human antiserum (USAMRIID) and secondary detection antibody goat anti-human conjugated to AlexaFluor 488 (1:2000 dilution; Invitrogen, Waltham, MA, USA). Percent infection was determined using the Cytation5 high-content imaging instrument, and data analysis was performed using the Gen 5.11 software (BioTek, Winooski, VT, USA). The percentage of viral inhibition was determined in comparison with infected, untreated cells, a dose–response curve was generated, and the area under the curve was reported.

### 2.9. Cytokine and Chemokine Analysis

Serum cytokine and chemokine levels were measured using the MILLIPLEX Premixed 32 Plex Mouse Cytokine/Chemokine Magnetic Bead Panel (Sigma-Aldrich, Burlington, MT, USA) according to the manufacturer’s protocol. Samples were analyzed on a Luminex MAGPIX System (ThermoFisher Scientific, Waltham, MA, USA) using the xPONENT 4.2 software (ThermoFisher Scientific, Waltham, MA, USA). The Median Fluorescent Intensity (MFI) of standard controls was plotted for each cytokine using a 5-parameter logistic curve-fitting method to calculate cytokine/chemokine concentrations in sera per manufacturer’s protocol.

### 2.10. Histopathology

Necropsies were performed on each mouse immediately following euthanasia in the USAMRIID biosafety level 4 laboratory. Tissues were fixed by immersion in 10% neutral buffered formalin (Valtech) and held in containment for a minimum of 21 days. Tissues were trimmed, processed, embedded in paraffin, cut by microtomy, stained with hematoxylin and eosin, cover-slipped, and screened.

### 2.11. In Situ Hybridization

Tissue sections were placed on positively charged slides and stained by ISH. To detect CHAPV genomic viral RNA in formalin-fixed embedded tissues, ISH was performed using the RNAscope 2.5 HD RED kit (Advanced Cell Diagnostics, Newark, CA, USA). ISH probes targeting nucleotides 392–1420 of CHAPV segment S (GenBank #NC_010562.1) were designed and synthesized by Advanced Cell Diagnostics (Cat #1291278-C1). Tissue sections were deparaffinized with xyless II (Valtech, Pottstown, PA, USA), followed by a series of ethanol washes and peroxidase blocking, and then heated in kit-provided antigen retrieval buffer and digested by kit-provided proteinase. Sections were exposed to ISH target probe pairs and incubated at 40 °C in a hybridization oven for 2 h. After rinsing with wash buffer, ISH signal was amplified using kit-provided Pre-amplifier and Amplifier conjugated to alkaline phosphatase and incubated with a Fast Red substrate solution for 10 min at room temperature. Sections were then stained with hematoxylin and eosin, air-dried, and cover-slipped. All slides were read blinded by a board-certified anatomic veterinary pathologist.

### 2.12. PCR

Serum samples were inactivated using a 3:1 ratio of TRIzol LS Reagent to sample (ThermoFisher, Waltham, MA, USA). A total of 300 µL of Trizoled material was purified using the EZ1 Virus Mini Kit v 2.0 (Qiagen, Germantown, MD, USA) using the EZ1 Advanced XL robot (Qiagen, Germantown, MD, USA) according to the manufacturer’s recommendations. The total elution volume was 150 µL. RT-qPCR was performed using TaqPath RT-qPCR Master Mix, CG (ThermoFisher, Waltham, MA, USA), on the QuantStudio DX (ThermoFisher, Waltham, MA, USA), with primers and probes targeting the L protein gene of the Chapare virus. The reference material was quantified using the QIAcuity Digital PCR System and the QIAcuity One-Step Viral RT-PCR Kit (Qiagen, Germantown, MD, USA) using the same RT-qPCR assay. Samples were run in technical triplicate, and concentrations were calculated using a standard curve of quantified reference material included on each run. Sample concentrations were reported in copies of target per mL and were based on extracted material. Concentrations that fell outside the linear range of the standard curve were extrapolated. The limit of detection of the assay based on the standard curve is approximately 10 copies/µL.

### 2.13. Sequencing

For sequence confirmation and contamination detection, each strain was subject to next-generation sequencing on the Illumina MiSeq platform (Illumina, Inc., San Diego, CA, USA). First, RNA from samples was extracted using the Purelink RNA mini kit (Invitrogen, Waltham, MA, USA). Isolated RNA was amplified using a sequence-independent, single-primer amplification (SISPA) protocol [19]. Sequencing libraries were prepared using the Illumina DNA Prep kit and sequenced on an Illumina MiSeq using a 600-cycle kit. Sequences were trimmed and quality-filtered with Trimmomatic (v.0.39) [20], then mapped to the CHAPV Strain 810419 L segment (EU260464) and S segment (EU260463) using SeqMan NGen (v.17.5.1. DNASTAR, Inc. Madison, WI, USA) and in-house scripts for sequence confirmation and SNP calling. Custom scripts were used to output allele frequencies at each position, and only SNPs above 50% frequency at positions with at least 20× depth were incorporated into the consensus. Each segment for each strain had a coverage greater than 99% (minimum depth: 20 bp) and an average depth of greater than 800×. There were no gaps in coverage observed internal to any segments, and all coding sequences are completely covered. Sequencing data was also assembled into contigs using ray (v.2.2) [21] and in-house scripts. Contigs were serially blasted against the nt database on multiple settings to exclude the presence of contaminating bacteria or viruses from the strains. Sequences were submitted to Genbank, and reference numbers are displayed in Table 1.

### 2.14. Statistical Analyses

Survival outcomes were compared against each group by Mantel–Cox tests. Weight loss and clinical outcomes were compared against each group by the Analysis of Variance (ANOVA) test. Cytokine data was compared across time points and against each group by ANOVA (Mixed-Effects). Statistical significance was determined by *p* ≤ 0.05. All statistical analyses were conducted in the Prism Software V9.5.1 (GraphPad, Boston, MA, USA).

### 2.15. Biosafety Considerations

All virus manipulation and in vivo experimentation were conducted in a biosafety level 4 (BSL-4) environment following all institutional biosafety Standard Operating Procedures (SOPs) and regulatory requirements and in compliance with the CDC’s Biosafety and Microbiological and Biomedical Laboratories (BMBL) guidelines. Animal experiments were conducted in the ABSL-4 facility and were approved by USAMRIID IACUC. Inactivated samples were transferred from the BSL-4 facility to BSL-2 laboratories following validated protocols approved by the Institutional Biosafety Committee at USAMRIID.

## 3. Results

### 3.1. CHAPV Is Partially Lethal in STAT1^-/-^ Mice

**Figure 1:**
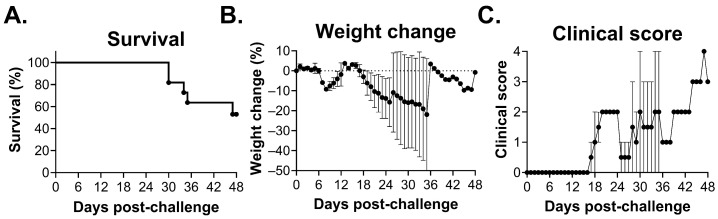
Wild-type CHAPV is partially lethal in STAT1-knockout mice. Six–ten-week-old STAT1^-/-^ mice were infected with 1000 pfu of CHAPV via the intraperitoneal route and monitored for (**A**) survival, (**B**) weight loss, and (**C**) clinical score for 48 days post-challenge. Data represents two experiments (*n* = 5–6 mice each). Error bars represent the standard error of the mean (SEM).

STAT1^-/-^ mice have been reported to be susceptible to infection by several arenaviruses, including Lassa virus and Machupo virus [12,22]. We hypothesized that STAT1^-/-^ mice might also be susceptible to CHAPV infection. Mice were infected with 1000 plaque-forming units (PFU) of CHAPV via the IP route and monitored for morbidity and mortality. Challenge resulted in partial lethality (defined by either death or euthanasia of moribund mice) of ~45% (mean time to death [MTD]: 35.17 ± 6.24) (Figure 1A). Disease was biphasic, with the first phase of disease occurring approximately 6–10 days post-challenge and resulting in body weight loss of approximately 10% (Figure 1B), but without any overt clinical signs of disease (Figure 1C). By day 13, mice had subsequently re-gained their weight back to the baseline, and at day 17 post-challenge, they entered the second phase of disease. The second phase was characterized by an average body weight loss of approximately 20% and severe clinical signs of disease including hunched posture and severe lethargy that progressed to neurological manifestations such as head tilt, circling, ataxia, and full or partial hind limb paralysis. These disease signs were followed by lethality between days 30 and 48 post-challenge. Data is reflection of two independent experiments, and we observed significant variation in weight loss and clinical scores, particularly in the second phase of disease. This is likely a reflection of the fact that individual mice progressed through disease at different rates, with some mice experiencing longer critical phases of disease prior to lethality and other mice experiencing delayed symptom onset, as late as 37 days post-challenge, followed by lethality.

### 3.2. Disease Progression Following CHAPV Infection of STAT1^-/-^ Mice

Serial sampling studies were performed to investigate the course of disease and pathogenesis following CHAPV infection in STAT1^-/-^ mice. Mice were challenged with 1000 pfu of CHAPV, and five mice per time point were euthanized, and the brain, kidneys, liver, lungs, spleen and serum were harvested for viral load analysis by plaque assay on days 0, 2, 4, 6, 8, 10, 12, 14, 16, 18, 20, 23, 28, and 35 days post-challenge. A similar biphasic disease course was observed, with the first phase occurring between days 5 and 10 post-challenge, characterized by 10% weight loss and no observable clinical signs of disease, and the second phase occurring 14 days post-challenge and onwards, characterized by severe weight loss and disease signs (Supplementary Figure S1).

#### 3.2.1. Infectious Viral Load

**Figure 2:**
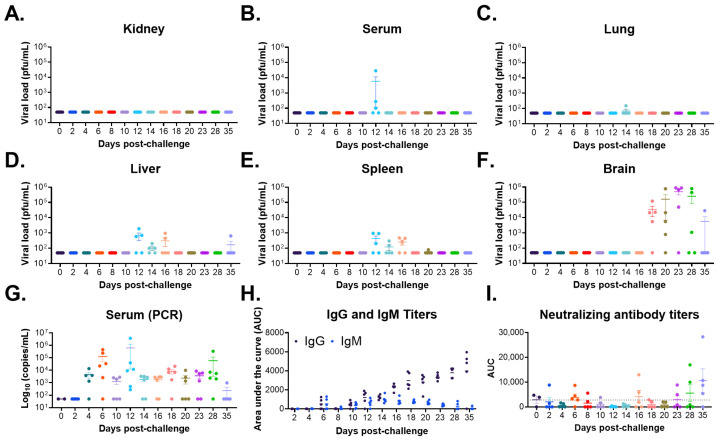
Disease progression of CHAPV in STAT1^-/-^ mice. Four–twelve-week-old STAT1^-/-^ mice were infected with 1000 pfu of CHAPV via the intraperitoneal route and tissue samples were harvested at the indicated time points following CHAPV infection. (**A**) The kidneys, (**B**) serum, (**C**) lungs, (**D**) liver, (**E**) spleen, and (**F**) brain were evaluated for infectious virus by plaque assay. (**G**) Serum was evaluated for CHAPV-specific genome copies by PCR. (**H**) IgG (purple) and IgM (blue)-specific antibodies and (**I**) neutralizing antibodies against CHAPV were assessed by ELISA and microneutralization assay, respectively. *N* = 5 mice per time point (*n* = 3 for day 0 only) are shown. Error bars present SEM.

At all tested time points, no infectious virus was observed in the kidneys of any sampled mouse (Figure 2A), and virus was detected in the serum (Figure 2B) and lungs (Figure 2C) of only 60% and 20% of mice at a single time point on day 12 and 14, respectively. Virus was detected transiently in the liver (Figure 2D) and spleen (Figure 2E) between days 12 and 16, although a single mouse also had the virus in the liver on day 35 post-challenge. Virus was first detected in the brain on day 18 post-challenge, reaching >10^4^ pfu/mL, and persisted until the end of the study (Figure 2F). Unlike the other tissues, the detection of virus in the brain was more uniform, whereas most mice (≥60%) at each time point displayed brain titers between days 18 and 35 post-challenge. Furthermore, viral titers in the brain were the highest of any tissue sampled at any time point. Appearance of infectious virus in the brain on day 18 (Figure 2F) closely proceeded with the initiation of the second, lethal phase of disease on day 14 (Supplementary Figure S1A) characterized by significant weight loss (>10% weight loss), severe clinical signs of disease (including numerous neurological signs, such as hind limb paralysis [partial and full], ataxia, heat tilt and circling), and mortality. Given the lack of detectable infectious virus in many of the tissues and the serum (excluding the brain) across time points, we further evaluated genomic copies in the sera of infected animals by PCR. This analysis revealed detectable genomic copies (10^3^–10^6^ copies/mL) in the serum at all time points tested (except for day 0 controls) at relatively equal levels over time (Figure 2G), although data was varied between individual mice at each time point.

#### 3.2.2. Antibody Titers and Neutralizing Response

We further assessed the development of CHAPV-specific IgM and IgG titers by ELISA (Figure 2H). IgG and IgM titers were observed starting day 6 with IgM titers peaking on day 16 and dropping to undetectable levels by day 35 while IgG titers continued to increase until the end of the study. Examination of the neutralizing antibody response following challenge revealed baseline levels of neutralization (relative to day 0, uninfected animals) at all time points through day 23 post-challenge, at which time neutralizing antibody responses increased in magnitude, although these differences were not significant relative to day 0 controls (Figure 2I). The presence of neutralizing antibody titers at later days post-challenge could explain the lack of detection of infectious virus in the serum at later days post-challenge.

#### 3.2.3. Anatomic Pathology in the Liver and Spleen

**Figure 3:**
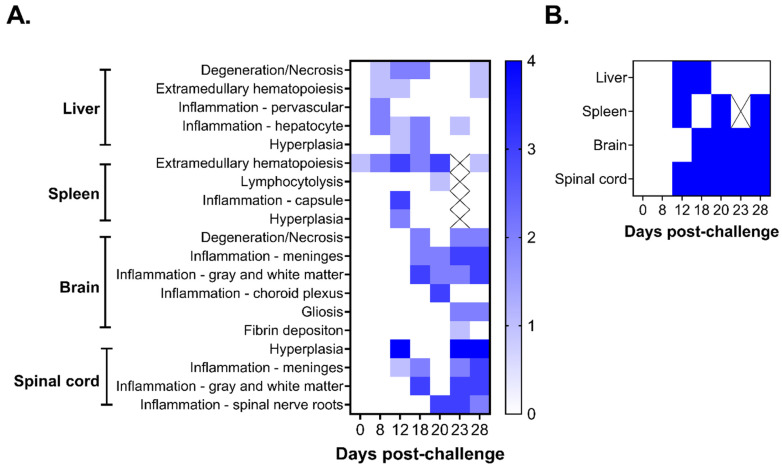
Pathology. Heat maps summarizing (**A**) microscopic and (**B**) ISH findings. In (**A**), 0 is no lesion detected; 1 is minimal, 0–10% of the section is affected; 2 is mild, 11–25% of the section is affected; 3 is moderate, 26–50% of the section is affected; and 4 is marked; >51% of the tissue is affected. In (**B**), white is no signal present and blue is signal present. One mouse was selected at random at each time point.

**Figure 4:**
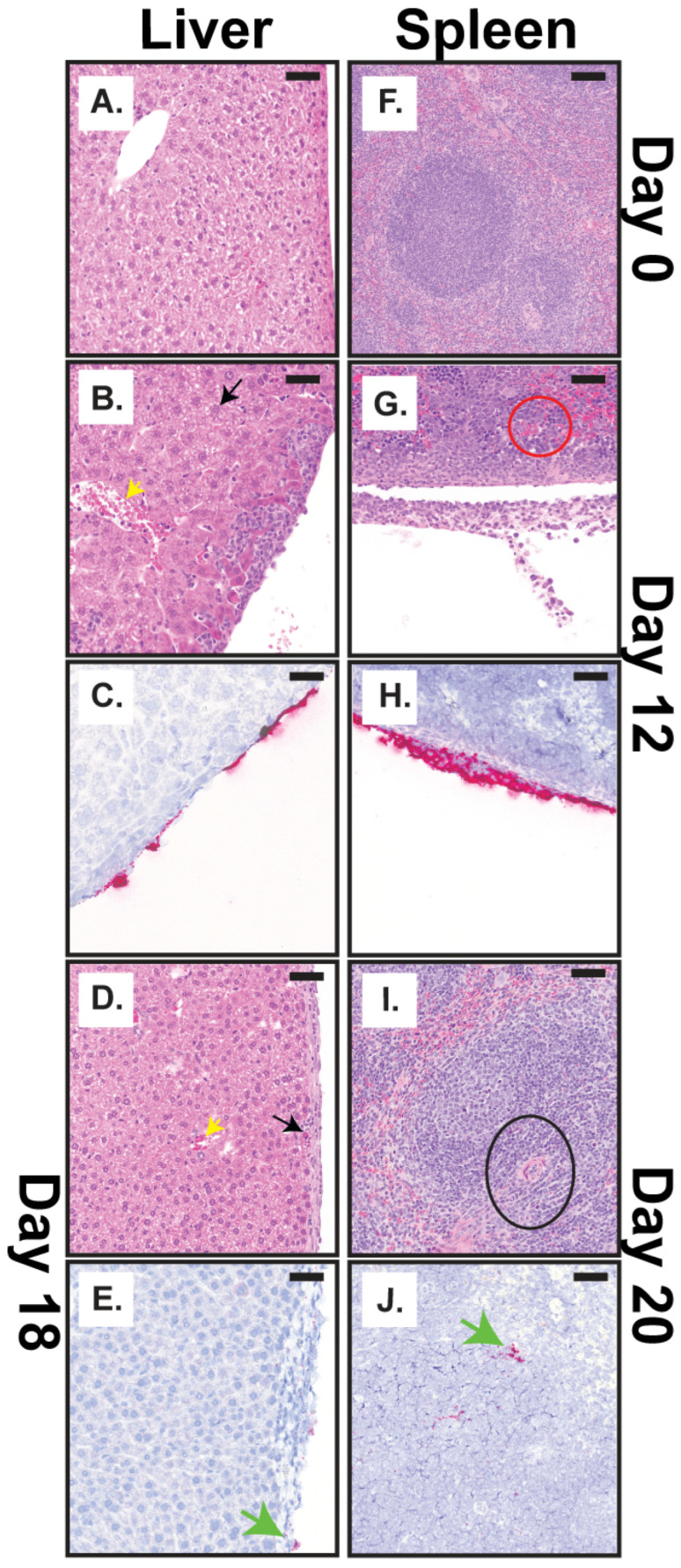
Pathologic findings in the spleen and liver. (**A**,**B**,**D**,**F**,**G**,**I**) Representative pictographs of microscopic findings by H&E and (**C**,**E**,**H**,**J**) ISH findings. (**A**–**E**) Liver and (**F**–**J**) spleen at (**A**,**F**) day 0, (**B**,**C**,**G**,**H**) day 12, (**D**,**E**) day 18, and (**I**,**J**) day 20. Scale bar is 50 µm. Black arrows represent hepatocellular degeneration and necrosis, yellow arrows represent the infiltration of macrophages and neutrophils, green arrows represent RNA in the hepatic capsule (**E**) and splenic white pulp (**J**), red circles represent expansion by macrophages and neutrophils, and black circles represent lymphocytolysis.

Histological and immunohistochemical changes in the liver, spleen, brain, and spinal cord throughout the course of disease were evaluated by hematoxylin and eosin (H&E) and in situ hybridization (ISH). The first significant microscopic findings following challenge occurred in the liver and the spleen. In the liver, on day 8, there was degeneration and necrosis of a few hepatocytes (black arrows) with multifocal, mild neutrophilic inflammation and mild expansion of perivascular areas and infiltration by small numbers of neutrophils and lymphocytes (yellow arrows) (Figure 3A and Supplementary Figure S2A), which increased in severity by day 12 post-challenge (Figure 3A and Figure 4A,B). By day 18, inflammation also occurred in the subcapsular areas and hepatic capsule with hepatocyte degeneration and low numbers of infiltrating neutrophils within the capsule. CHAPV-specific RNA was also detected in the liver on days 12 and 18 in overlapping locations where inflammation was observed on the serosal surface and in the hepatic capsule, respectively, although RNA detection was minimal at day 18 (Figure 3B and Figure 4C,E). In general, microscopic findings in the liver were nonexistent on days 20–23 with minimal degeneration and necrosis (Figure 3A) and a lack of detectable CHAPV-specific RNA (Figure 3B). However, we did observe some hepatocellular degeneration, necrosis, and neutrophil infiltration on day 28 in the liver (Supplementary Figure S2B), though inflammation was minimal.

In the spleen, some mild expansion of the red pulp by extramedullary hematopoiesis (EMH) was observed in unchallenged animals (Figure 3A and Figure 4F). Although EMH is a common background finding in mice [23,24], it increased in severity through day 20 post-challenge (Figure 3A). By day 12 post-challenge, a moderate number of neutrophils, macrophages, and lymphocytes expanded the serosal surface and splenic capsule (red circle) with occasional hyperplasia (e.g., increased cell number) of mesothelial cells (Figure 3A and Figure 4G) followed by multifocal lymphocytolysis (disintegration of lymphocytes) within germinal centers of lymphoid follicles (black circle) within the white pulp day 20 post-challenge (Figure 3A and Figure 4I). Detection of CHAPV-specific RNA in the spleen was inconsistent between individual mice across time points but was generally observed on days 12 to 20 post-challenge in areas of inflammation, particularly on the serosal surface and in the splenic white pulp (green arrows) with reducing RNA detection after day 12 and minimal histological lesions or viral RNA detected after day 20 (Figure 3B and Figure 4H,J).

#### 3.2.4. Anatomic Pathology in the Brain and Spinal Cord

**Figure 5:**
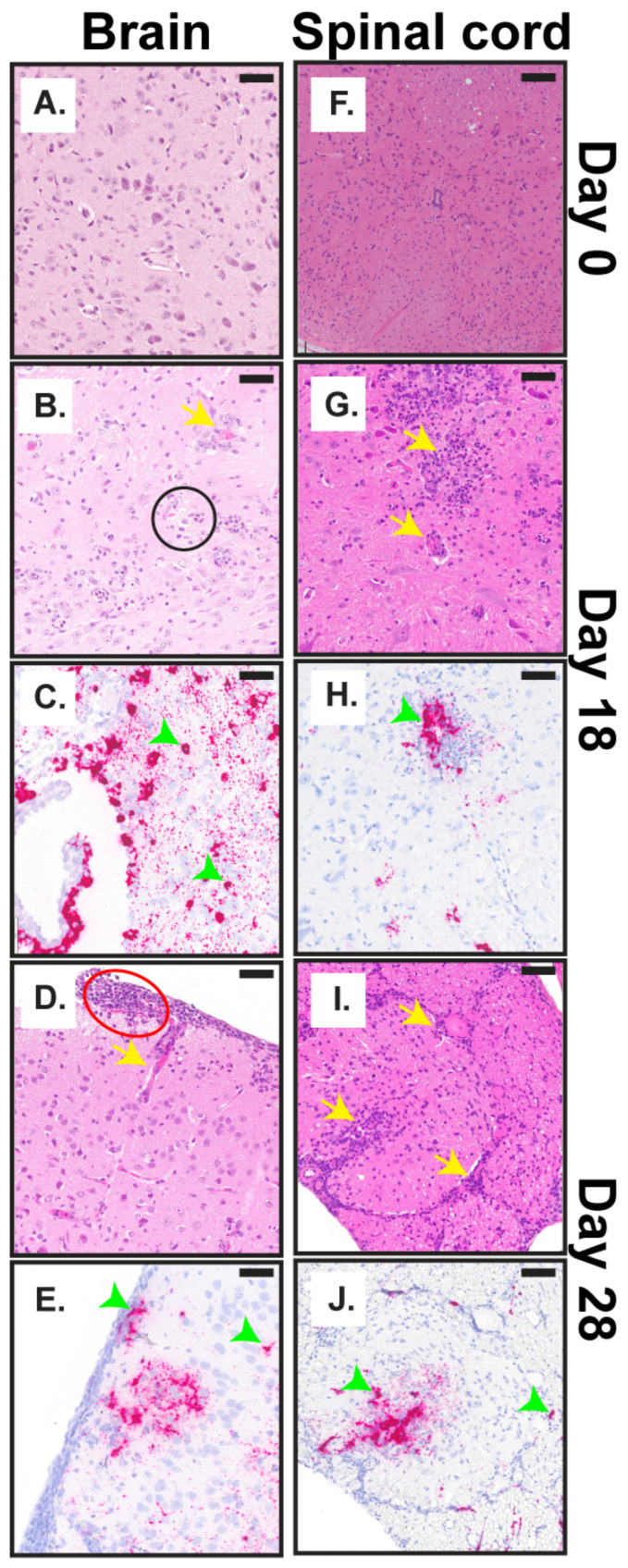
Pathologic findings in the brain and spinal cord. (**A**,**B**,**D**,**F**,**G**,**I**) Representative pictographs of microscopic findings by H&E and (**C**,**E**,**H**,**J**) ISH findings. (**A**–**E**) The brain and (**F**–**J**) spinal cord on (**A**,**F**) day 0, (**B**,**C**,**G**,**H**) day 18, (**D**,**E**,**I**,**J**) and day 28. Scale bar is 50 µm. Black circles represent multifocal vacuolation, red circles represent expansion of the meninges, yellow arrows represent infiltration by neutrophils, and green arrows represent viral RNA in neurons and at sites of inflammation.

No microscopic lesions were detected in the brain until day 18 post-challenge (Figure 3A), at which point infiltration of moderate numbers of neutrophils in the gray and white matter at all levels of the brain from the olfactory bulbs to the cerebellum was observed (Figure 5A,B). Specifically, the neuropil, the dense network of axons, dendrites, and glial cells in the central nervous system (CNS), was infiltrated by neutrophils (yellow arrows), and multifocal vacuolation (black circles) was observed (Figure 5B). Additionally, within the affected neuropils, spongiosis (rarefaction of the neuropil), karyorrhectic cellular debris (neural and/or glial cell death), and the occasional microglial cells with moderate expansion of the meninges by neutrophils, macrophages, and lymphocytes were observed (Figure 3A and Supplementary Figure S2C, red circles). CHAPV-specific RNA was also detected in the brain by day 18, primarily present in the neurons and glial cells (Figure 3B and Figure 5C, green arrows). Histological findings progressed in severity overtime. By day 20, multifocal necrosis (black arrows) was observed in the epithelium, and moderate numbers of neutrophils, macrophages, and lymphocytes infiltrated (yellow arrows) and expanded the choroid plexus, a network of capillaries in the brain’s ventricles that produce cerebrospinal fluid (Supplementary Figure S2D), and viral RNA was detected in epithelial cells and at sites of inflammation (Supplementary Figure S2D, green arrows). Infiltration and expansion by neutrophils, macrophages and lymphocytes in the meninges (Supplementary Figure S2F) and neuropil (Supplementary Figure S2F) were also noted on day 23. By day 28, there was neutrophilic infiltration of the white and gray matter with degeneration of the neuropil and proliferation of microglial cells (gliosis), expansion of the meninges by inflammatory cells, and fibrin deposition (Figure 3A and Figure 5D). Similarly, the detection of viral RNA increased from day 18 to day 28, and viral RNA was widely dispersed throughout all areas of the brain particularly in areas of meningeal inflammation and within neurons (Figure 5E).

Microscopic lesions in the spinal cord were detected by day 12 post-challenge with focal areas of inflammation in the meninges composed of small numbers of neutrophils, macrophages, and lymphocytes and diffuse marked hyperplasia of granulocytic precursor cells in the vertebral bone marrow (Figure 3A). Significant inflammation, including moderate numbers of infiltrating neutrophils and macrophages (yellow arrows) within the gray and white matter and expansion of perivascular spaces by neutrophils, was observed on day 18 post-challenge and was present within the nervous tissue of the spinal cord, parallelling findings in the brain at the same time point. CHAPV-specific RNA was also detected in the spinal cord starting day 12 with moderate staining at sites of inflammation, and viral RNA was present within the neuropil itself (Figure 3B and Figure 5H, green arrows). As observed in the brain, severity of histological findings in the spinal cord increased over time. Neutrophilic inflammation extended into adjacent spinal nerve roots with moderate inflammation in the spinal cord neuropil and nerve roots and infiltration by neutrophils and macrophages by day 20 and 23, respectively (Figure 3A and Supplementary Figure S2H). Viral RNA was also detected within the spinal nerve roots on day 20 (Supplementary Figure S2I, green arrows). At 28 days post-challenge, there was continued neutrophilic inflammation in the gray and white matter of the spinal cord, meninges, and adjacent nerve roots (yellow arrows) together with marked hyperplasia of the granulocytic cell precursors in the vertebral bone marrow (Figure 3A and Figure 5I). Similarly, on day 28, we observed diffuse viral RNA throughout the spinal cord gray and white matter (Figure 5J).

### 3.3. Adaptation of CHAPV in STAT1^-/-^ Mice

**Figure 6:**
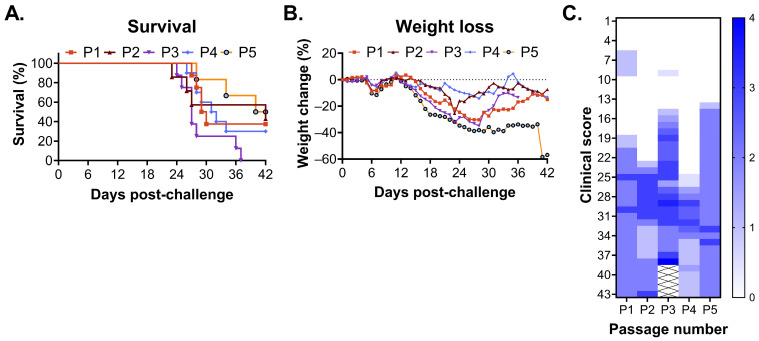
CHAPV passaged in the brain of STAT1^-/-^ mice results in a fully lethal infection. Six–ten-week-old STAT1^-/-^ mice (*n* = 6–8) were infected with 1000 pfu of CHAPV passaged one [P1], two [P2], three [P3], four [P4], or five [P5] times in the brains of STAT1 KO mice via the intraperitoneal route and monitored for survival (**A**), weight change (**B**), and clinical score (**C**) for 42 days post-challenge. Data is representative of a single experiment of *n* = 8 (P1, P2, P3, P5) or *n* = 10 (P4) mice.

With the aim of improving disease severity and lethality of CHAPV in STAT1^-/-^ mice, the brain tissue from STAT1^-/-^ mice challenged with CHAPV was sequentially passaged. For the first passage, STAT1^-/-^ mice were challenged with CHAPV by the IP route, and the brain tissue was collected from four mice between days 27 and 30 post-challenge. Decisions to euthanize and harvest the brain tissue were made when mice were exhibiting severe clinical signs of disease (lethargy, hunched posture, weight loss, neurological manifestations such as ataxia, head tilt, and paralysis). The brain tissue was homogenized and clarified of large debris by centrifugation, and viral titer was assessed by plaque assay. The brain tissue with the highest viral titer from one to two mice was combined, and for each subsequent passage, the brain homogenate was used to challenge naïve STAT1^-/-^ mice without purification or isolation. At each additional passage, one to two mice were harvested for the brain tissue at individual time points between days 27 and 38 as above. This serial passaging in STAT1^-/-^ mice was performed five times. To monitor adaptation during passaging, we evaluated small groups of STAT1^-/-^ mice (*n* = 5–7 mice) for weight loss, clinical score, and mortality for 42 days post-challenge (Figure 6). After passages 1 and 2, we observed approximately 60% lethality with severe weight loss (approximately 40%) and clinical scores of disease that looked similar to wild-type CHAPV (Figure 1). By passage 3, we observed 100% lethality within 37 days post-challenge and clinical signs of disease that progressed rapidly on day 13 post-challenge (Figure 6A–C). Further passaging of the virus (e.g., passage 4 and 5) resulted in the loss of lethality (Figure 6A) and, in the case of passage 4, lesser weight loss (Figure 6B) and delayed symptom onset (Figure 6C). Based on these studies, we grew a virus stock of the passage 3 challenge material in vitro on VeroE6 cells, hereafter termed mouse-adapted (ma) CHAPV. We subsequently challenged *n* = 8 STAT1^-/-^ mice with ma-CHAPV to confirm lethality of our cell-culture-grown ma-CHAPV stock. As expected, we observed a biphasic disease course with 90–100% lethality (Supplementary Figure S3).

### 3.4. Disease Progression Following ma-CHAPV Infection of STAT1^-/-^ Mice

#### 3.4.1. Infectious Virus Load

Serial sampling studies were performed to investigate changes in disease progression and pathogenesis following mouse adaptation. Mice were challenged by the IP route with 1000 pfu of ma-CHAPV, and on days 4, 8, 12, 18, 20, 23, 29, and 36 days post-challenge, two to four mice at each time point were euthanized, and the brain, kidneys, lungs, liver, spleen, and serum were harvested for analysis of infectious viral load by plaque assay. Similar to non-passaged, wild-type CHAPV, a biphasic disease course was observed with the first phase resulting in ~10% weight loss and no observable clinical signs of disease 3–10 days post-challenge followed by the second phase 13 days onward characterized by severe weight loss (>20% weight loss), clinical signs of disease and mortality (Supplementary Figure S4). As before, individual mice progressed through disease at different rates, resulting in variable group weight loss and clinical scores throughout the course of disease. We also observed similar clinical signs of disease as compared to infection with wt-CHAPV, including paralysis (full and partial), ataxia, head tilt, and circling.

**Figure 7:**
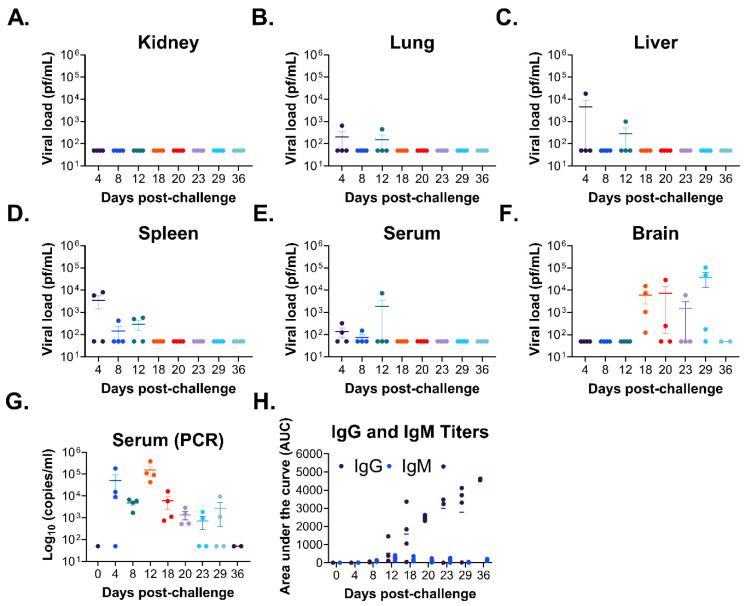
Disease progression of ma-CHAPV in STAT1^-/-^ mice. Four–twelve-week-old STAT1^-/-^ mice were infected with 1000 pfu of ma-CHAPV via the intraperitoneal route and tissue samples were harvested at the indicated time points following CHAPV infection. The kidneys (**A**), lungs (**B**), liver (**C**), spleen (**D**), serum (**E**), and brain (**F**) were evaluated for infectious virus by plaque assay. (**G**) CHAPV-specific genome copies in the serum were evaluated by PCR. (**H**) IgG (purple) and IgM (blue)-specific antibodies were assessed by ELISA. *N* = 4 per time point (*n* = 1 and *n* = 2 for day 0 and 36 for serum PCR analysis, respectively).

Similar to the non-passaged wild-type CHAPV, no infectious virus was observed in the kidneys of any mouse (Figure 7A) at any time point tested. Virus in the lungs (Figure 7B), liver (Figure 7C), spleen (Figure 7D), and serum (Figure 7E) was detected 4 days post-challenge and persisted until day 12 post-challenge. However, titers were inconsistent across mice, with only 25–50% of mice with detectable virus in these tissues. Virus in the brain (on average ≥10^4^) was first observed on day 18 post-challenge and persisted until day 29 post-challenge (Figure 7F). Infectious virus in the brain was more uniformly detected across mice on 18–29 days post-challenge. CHAPV-specific genome copies were detected in the serum by PCR, with varied levels across mice, as early as 4 days post-challenge, peaking at day 12 post-challenge and persisting through day 29 post-challenge (Figure 7G).

#### 3.4.2. Antibody Titers and Neutralizing Response

The IgG- and IgM-specific antibody response was assessed by ELISA **(**Figure 7H). Overall, IgM-specific antibodies were generally lower than IgG-specific antibodies. IgG- and IgM-specific antibodies were first detected on day 12 post-challenge with IgM-specific antibodies peaking on day 18–20 post-challenge and IgG-specific antibodies continuing to increase until the end of the study. Unfortunately, we did not have enough serum to evaluate neutralizing antibody titers.

#### 3.4.3. Cytokine and Chemokine Analysis

**Figure 8:**
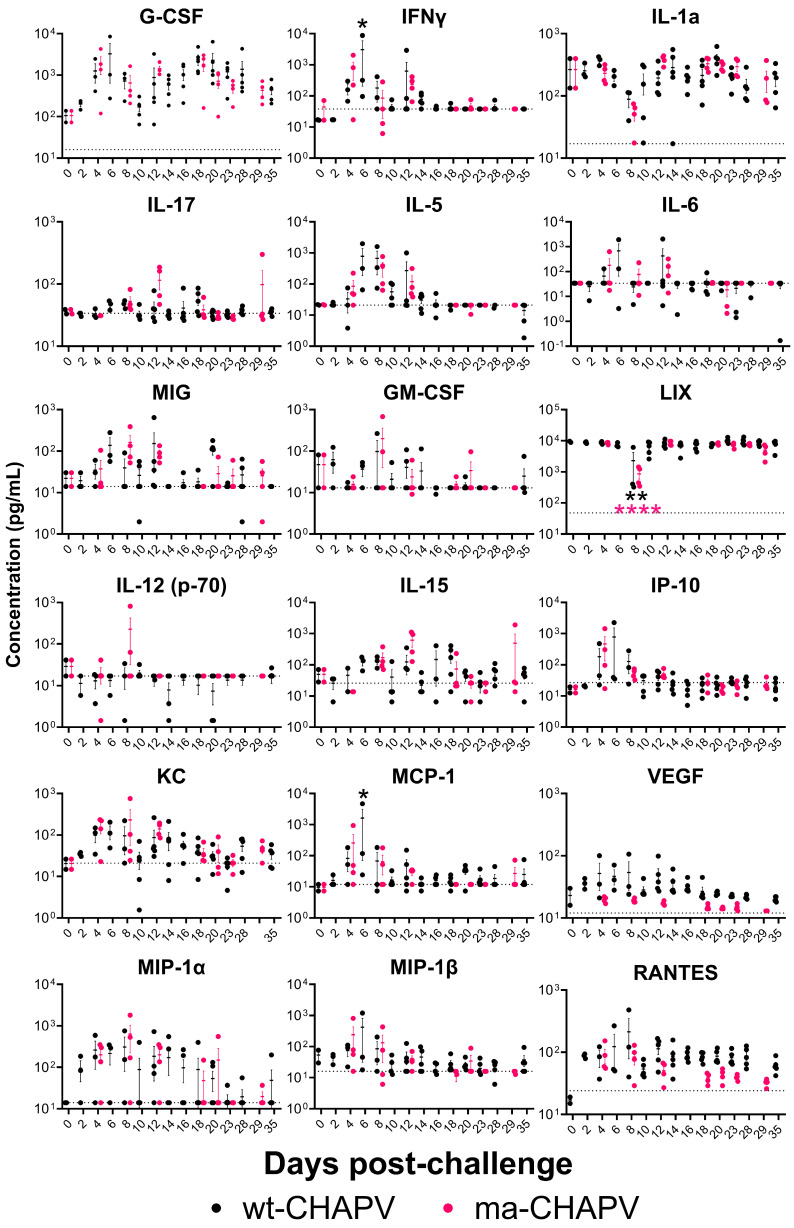
Cytokine and chemokine analysis. Levels of 18 different cytokines and chemokines in serum of STAT1^-/-^ mice challenged with either wt-CHAPV (black) or ma-CHAPV (pink) were evaluated at various times post-infection (*n* = 2–5 mice per time point) and concentration (pg/mL) was plotted. Mean is plotted and error bars are SEM. Dashed line indicates MFI of a blank sample from each cytokine. Cytokine/chemokine responses analyzed by ANOVA. Statistical significance is represented by * *p* ≤ 0.05, ** *p* ≤ 0.01, and **** *p* ≤ 0.001. Significance is color-coded to refer to group and is relative to baseline (day 0).

To further evaluate differences in the pathogenesis of disease caused by wild-type and ma-CHAPV, cytokine and chemokine levels were analyzed in sera from CHAPV-infected STAT1^-/-^ mice using samples from serial sacrifice studies (Figure 8). wt-CHAPV and ma-CHAPV infection of STAT1^-/-^ mice resulted in the production of multiple inflammatory cytokines and chemokines. The levels of granulocyte colony-stimulating factor (G-CSF), IFNγ, interleukin 5 (IL-5), IL-6 monokine induced by gamma interferon (MIG, CXCL9), granulocyte–macrophage colony-stimulating factor (GM-CSF), IL-15, interferon-gamma inducible protein 10 (IP-10, CXCL10), keratinocyte chemoattractant (KC, CXCL1), monocyte chemoattractant protein 1 (MCP-1, CCL2), macrophage inflammatory protein 1 alpha (MIP-1α, CCL3), MIP-1β (CCL4), and chemokine C-C motif ligand 5 (CCL5; e.g., RANTES) were all variably elevated relative to day 0 controls between days 2 and 16 post-challenge. Interestingly, reductions in cytokine responses for IL-1α and lipopolysaccharide-induced CXC chemokine (LIX, CXCL5) were observed between days 4 and 12 post-challenge upon infection with either wt-CHAPV or ma-CHAPV. Several cytokines/chemokines were increasingly elevated in mice challenged with ma-CHAPV compared to those challenged with wt-CHAPV, including IL-12(p70), IL-15, IL-17, KC, and MIP-1α while the inverse was true for IL-6, MCP-1, VEGF, and RANTES.

#### 3.4.4. Sequencing of CHAPV and ma-CHAPV

Stocks of wild-type and maCHAPV were sequenced and compared to ascertain genetic differences between the stocks. Surprisingly, no mutations in the large (L) segment were detected. However, one non-synonymous mutation in the nucleocapsid protein was observed in the mouse-adapted stock relative to the wild-type stock. This mutation resulted in a change from T to A (Table 2) and was observed at a frequency of 86.93%.

## 4. Discussion

To date, only two animal models for CHAPV have been described and utilize Strain 13 guinea pigs and cynomolgus macaques [8,9]. Although the Strain 13 guinea pig model results in fully lethal disease, numerous factors limit the use of this model, including the fact that Strain 13 guinea pigs are less commonly available and require specialized facilities for their care, have a potential unexplained sex bias, and do not fully recapitulate many aspects of human disease, including neurological manifestations. Conversely, the cynomolgus macaque model is not fully lethal, and NHPs are associated with significant logistical, financial, and ethical limitations that severely limit their use. No other animal models for CHAPV have been described, underscoring the need for additional small-animal models to enable a greater understanding of CHAPV pathogenesis. Such models will also serve as tools for antiviral therapeutic discovery, for which none currently exist. In this study, STAT1^-/-^ mice were infected with CHAPV, resulting in the first described lethal small-animal model for this poorly understood arenavirus. Previous studies have posited that type I IFN responses may be crucial for the control of arenavirus infection [25]. Both the arenavirus nucleoprotein (NP) and RING-finger Z protein have been described as type I IFN antagonists that contribute to general immunosuppression following arenavirus infection [25,26,27,28]. Specifically, the Z protein of new world arenaviruses, including CHAPV, can bind to the retinoic acid inducible gene I (RIG-I) and MDA5 and suppress the production of IFN-β [26] while NP can interfere with the activation of IRF3 and inhibit the IFN-I response [25,27,28]. In line with this data, a wealth of studies in mice have shown that a functional innate immune system with a productive IFN response is essential to control and clear arenavirus infection [12,17,22,29,30,31,32,33,34]. A STAT1^-/-^ mouse model has been reported to be susceptible to MACV infection, a NW arenavirus closely related to CHAPV [12]. Our findings are in line with these reports and confirm that STAT1^-/-^ mice are also susceptible to CHAPV infection, with a similar disease course.

The model described here parallels the biphasic disease course observed in some animal models of NW arenavirus infection, including the MACV non-human primate model and our recently described MACV STAT1^-/-^ mouse model [11,13,14,15,16,18]. Few animal models of CHAPV infection have been described. However, cynomolgus macaques, which display a non-lethal infection, share some commonalties with the STAT1^-/-^ model, including the fact that they display neurological signs, including ataxia and persistent tumors, following challenge with CHAPV. Little is known regarding the course of disease following human infection given the limited number of cases; however, late neurological syndrome is a common complication in humans following CHAPV infection, which can be lethal or can result in neurological complications that persist for several months [3]. Thus, findings in the STAT1^-/-^ model recapitulate the disease course typically observed during human infection, including the progression from initial febrile illness to neurologic disease [1,2,3]. As such, this murine model could be a valuable tool to further study the mechanistic underpinnings associated with the establishment of neurological disease caused by CHAPV. However, immunocompromised mouse models, while essential for providing small-animal models where no small-animal immunocompetent models exist, are limited by a lack of mature, functional immune systems and poor modeling of complex pathologies that could impact results, particularly for antiviral efficacy testing and evaluation. Continued optimization of this model is required to effectively utilize it for medical countermeasure testing. Additionally, further examination of CHAPV disease in additional animal species, particularly those with intact immune responses, and how the STAT1^-/-^ model can complement these models are of continued interest. Although wild-type CHAPV was not fully lethal in initial experiments, a novel mouse-adapted variant capable of causing severe disease and mortality was generated. Mouse adaptation of CHAPV resulted in the accelerated dissemination of the virus to the liver and spleen, with infectious virus detected on day 4 post-challenge with ma-CHAPV (Figure 7) compared to detection on day 12 post-challenge with wild-type CHAPV (Figure 3). However, dissemination of the virus to the brain occurred at similar times post-challenge with both CHAPV isolates. These findings are not surprising given the similar rates of disease progression in both studies (Supplementary Figures S1 and S4). We observed only one mutation in the NP of ma-CHAPV compared to the wild-type isolate (Table 2). Similarly, our ma-MACV isolate only had two mutations in the NP compared to the wild-type isolate [18]. The arenavirus NP is a multi-functional protein with roles in viral replication and transcription, infectious particle production and packaging, and antagonism of inflammatory responses, and as such, a mutation in the NP could impact any number of viral pathways or virus–cell interactions. Further mechanistic studies will be required to understand the enhanced lethality associated with ma-CHAPV and the functional relevance of this mutation.

We completed a histopathological examination of multiple tissues, including the liver, spleen, brain, and spinal cord throughout the course of disease following CHAPV challenge (Figure 3, Figure 4 and Figure 5, Supplementary Figure S2). Overall, our pathological investigations showed that the most consistent findings occurred in the brain and spinal cord. The lesions in these tissues were characterized by infiltration of inflammatory cells into multiple areas of the brain and spinal cord, including the neuropil, meninges, choroid plexus, and spinal nerve roots, with degeneration of the underlying neuropil and proliferation of microglial cells. The microscopic lesions progressively became more severe and continued through day 28. Lesions in the liver and spleen were observed at earlier times post-challenge (days 8–20 post-challenge) but were inconsistent and most of the lesions were centered on the capsule and parenchyma just subjacent to the capsule. These findings are in line with the viral load patterns observed in these tissues (Figure 2). These results show a relationship between development of infectious virus (Figure 2 and Supplementary Figure S2) and histopathological findings in the brain and spinal cord (Figure 5 and Supplementary Figure S2) and initiation of the second, lethal phase of disease. Taken together, these findings suggest that dissemination of virus to the brain cause the severe disease and neurological manifestations that subsequently lead to mortality in STAT1^-/-^ mice. However, these results are limited by the use of a single infection route and dose (e.g., intraperitoneal at 1000 pfu), which have significant implications on the speed of disease progression, tissue tropism, severity of pathology, and the nature of the immune response in animal models [35]. This was shown to be true for Machupo virus in the STAT1^-/-^ model, where different infection routes had significant impacts on the course of disease [12,18]. Thus, a deeper examination of the impact of alternative infection routes and doses of infection on mortality, disease progression, and pathology in this model should be undertaken in future efforts.

Histopathological changes following CHAPV challenge to date have been limited, particularly in humans. Clinical findings in fatal cases reveal pulmonary edema, severe gastrointestinal hemorrhage, vascular leakage, shock, necrotizing lesions in the liver and spleen parenchyma, encephalitis and neuro-inflammation [1,3]. Recently, the disease progression in Strain 13 guinea pigs and cynomolgus macaques following CHAPV challenge has been described [8,9]. Strain 13 guinea pigs showed systemic infection similar to guinea pig models of LASV infection [36,37] with viral RNA in all tissues tested (lymph node, liver, spleen, kidney, lung, heart, pancreas, urinary bladder, gastrointestinal tract, and brain) [8], while in cynomolgus macaques, viremia was detectable only for the first week following challenge and viral RNA persisted in many tissues throughout the body at day 35 post-challenge [9]. Histopathology findings in Strain 13 guinea pigs and cynomolgus macaques noted significant pathology in the gastrointestinal tract with necrotizing hepatitis, but these findings were only evident for the single cynomolgus macaque that succumbed to challenge (out of four animals) [8,9]. Strain 13 guinea pigs also exhibited mild gliosis and positive immunohistochemistry staining in the brainstem of one animal. Here, CHAPV was detected in all tissues tested, but was characterized by more severe pathology in the brain and spinal cord that contributed to severe disease and mortality, a finding that was not described in Strain 13 guinea pigs and minimally in cynomolgus macaques. This likely reflects a difference in disease progression observed in these models versus STAT1^-/-^ mice, whereas STAT1^-/-^ mice exhibit delayed times to death associated with neurological disease and Strain 13 guinea pig disease is characterized primarily by hemorrhagic manifestations. The lack of an intact immune system by STAT1^-/-^ could skew infection towards neurological disease and guinea pigs towards hemorrhagic disease. Interestingly, we recently utilized similar methods to establish a lethal STAT1^-/-^ mouse model of MACV disease and plot the natural history of disease in this model [18]. Tissue adaptation in the brains of MACV-challenge mice resulted in a mouse-adapted stock of MACV that was fully lethal in STAT1^-/-^ mice. Furthermore, clinical disease and pathology observed in this study mirror what we have observed following Machupo virus infection in this STAT1^-/-^ mouse model [18] and in Hartley guinea pigs [10], in which initial systemic infection is followed by delayed neurological disease and death. Similar findings have been described in guinea pigs following Junin virus (JUNV) infection. Infection with hemorrhagic causing JUNV strains results in necrosis of spleen and lymph nodes with death in the second week following infection while infection with neurological causing strains results in viral dissemination to the brain and paralysis with death in the third and fourth weeks following infection [38]. Interestingly, aspects of JUNV disease following infection with either hemorrhagic or neurological causing strains recapitulate aspects of the first and second phase of CHAPV disease observed in STAT1^-/-^ mice described here, respectively.

Nothing has been done to characterize the inflammatory response in humans following CHAPV infection. The data reported here suggest dysregulated cytokine/chemokine responses primarily occurring during the first phase of disease (days 6–10 post-challenge), with some responses continuing through day 18, and early dysregulated inflammatory responses could trigger disease progression observed later in infection. Increased KC has been shown to induce pathogenic effects following viral infection [39]. The high levels of MCP-1, IP-10, IL-10, G-CSF, IFNy, and Il-6 have been linked to the pathogenesis of several viral infections, including chikungunya and COVID-19, and the levels of IL-6 and G-CSF have been correlated with severe human disease for related arenaviruses including JUNV [40,41,42,43,44,45]. Generally, mice challenged with ma-CHAPV had a stronger inflammatory response compared to wild-type-CHAPV, particularly for IL-12p70, IL-15, IL-17, KC, and MIP-1α (Figure 8), although this was not significant. This response was associated with delayed viral control (Figure 7) and more severe disease (Figure 6), suggesting that these responses may contribute to the distinct disease outcome. Furthermore, these aberrant inflammatory responses could trigger neuro-inflammatory responses and the attraction of more immune cells into the brain. STAT1^-/-^ mice exhibit defects in type I, II, and III IFN signaling; therefore, it is likely that the cytokine and chemokine data reported here may not be wholly representative of the inflammatory response following infection in humans. However, the data reported here lays the foundation for future studies evaluating the cytokine and chemokine response to CHAPV infection. More studies looking at these responses in additional animal models and following human infection are required.

In conclusion, ma-CHAPV represents a significant advancement for research into CHAPV by enabling in vivo evaluation of the virus in a small-animal model that recapitulates many aspects of severe cases of human CHHV. However, immunocompromised models have significant limitations that should be considered regarding translational relevance for medical countermeasure development. Therefore, further evaluations of the model are required to understand how it may be effectively used for antiviral therapeutic efficacy testing and development and in combination with previously published CHAPV animal models. A logical next step of this work would be to evaluate the susceptibility of immunocompetent mouse strains to ma-CHAPV, which could provide a more relevant, tractable, and translatable animal model. Additional efforts should also seek to further our understanding of the mechanistic underpinnings associated with neurological disease in this model, which can serve as a useful tool for evaluating medical countermeasures aimed at alleviating neurological manifestations.

## Figures and Tables

**Table 1:** GenBank references. wt-CHAPV strain is derived from strain 810419 (GenBank EU260464/EU260463) and passaged once in VeroE6 cells to obtain a viral stock (GenBank numbers below). ma-CHAPV was derived from the challenge material (e.g., brain homogenates from challenged naïve STAT1^-/-^ mice observing clinical signs of disease) from passage 3 animals.

GenBank Number	Virus	Segment
PX873550	ma-CHAPV	L segment
PX873551	ma-CHAPV	S segment
PX873552	wt-CHAPV derived from (strain 810419)	L segment
PX873553	wt-CHAPV (derived from strain 810419)	S segment

**Table 2:** Sequencing data.

Virus	Type	Base	SNP	Codon	Feature	Frequency
ma-CHAPV	Mutation	A	G	T (ACT) @542 A (GCT)	Nucleocapsid protein	86.93

## Data Availability

Data related to this paper may be requested from the authors.

## Supplementary Materials

The following supporting information can be downloaded at: https://www.mdpi.com/article/10.3390/v18030388/s1, Supplementary Figure S1: Weight change and clinical score following challenge with wt-CHAPV in STAT1^-/-^ mice; Supplementary Figure S2: Pathological findings in the liver, brain, and spinal cord; Supplementary Figure S3: Survival, weight change, and clinical score following challenge with ma-CHAPV in STAT1^-/-^ mice; and Supplementary Figure S4: Weight change and clinical score following challenge with ma-CHAPV in STAT1^-/-^ mice.

## Abbreviations

The following abbreviations are used in this manuscript:

CHAPV	Chapare virus
CHHF	Chapare hemorrhagic fever
STAT1^-/-^	Signal transducer and activator of transcription 1-knockout mice
MACV	Machupo virus
ATCC	American Type Culture Collection
DMEM	Dulbecco’s Modified Eagle Medium
FBS	Fetal bovine serum
MOI	Multiplicity of infection
IP	Intraperitoneal
PFU	Plaque-forming units
HRP	Horseradish peroxidase
MFI	Mean fluorescent intensity
ANOVA	Analysis of Variance
SEM	Standard error of the mean
BSL-4	Biosafety level 4
SOP	Standard Operating Procedure
BMBL	CDCs Biosafety and Microbiological and Biomedical Laboratories guidelines
ISH	In situ hybridization
H&E	Hematoxylin and eosin
CNS	Central nervous system
G-CSF	Granulocyte colony-stimulating factor
IL	Interleukin
MIG	Monokine induced by gamma interferon
GM-CSF	Granulocyte–macrophage colony-stimulating factor
IP-10	Interferon-gamma inducible protein 10
KC	Keratinocyte chemoattractant
MCP-1	Monocyte chemoattractant protein 1
MIP	Macrophage inflammatory protein
CCL5	Chemokine C-C motif ligand (RANTES)
LIX	Lipopolysaccharide-induced CXC chemokine
RIG-I	Retinoic acid inducible gene I
NP	Nucleoprotein
JUNV	Junin virus
