# Supplementary material for: A STAT1-Knockout Mouse Model for Chapare Virus Infection and Pathogenesis
Source: Viruses. 2026 Mar 20;18(3):388. doi: 10.3390/v18030388 (PMC13030867; doi:10.3390/v18030388)
Supplement: Supplementary file 1 [file viruses-18-00388-s001.zip › Supplemental Files.pdf]

Supplementary Figure S1. Weight change and clinical score following challenge with wt-CHAPV in STAT1$^{-/-}$ mice. Four- to twelve-week-old STAT1 knockout mice were infected with 1000 pfu of wt-CHAPV via the intraperitoneal route and monitored daily for (A) weight change and (B) clinical score for 35 days post-challenge. Error bars represent standard error of the mean (SEM). Data is representative of one study of *n* = 72 mice with *n* = 5 mice harvested days 2, 4, 6, 8, 10, 12, 14, 16, 18, 20, 23, 28, and 35 post-challenge. Mice were separated into four pans of females (*n* = 2, 10, 10, 10 mice per pan) and four pans of males (*n* = 10 per pan), group weighed, and pan weights reported.

Supplementary Figure S2. Pathological findings in liver, brain, and spinal cord. (A-F, H) Representative pictographs of microscopic findings by H&E and (G, I) ISH findings. (A-B) Liver, (C-G) brain, and (H-I) spinal cord at (A) day 8, (C) day 18, (D-E, H-I) day 20, (F-G) day 23, and (B) day 28. Scale bar is 50 µm. Red circles represent expansion of the meninges, yellow arrows represent infiltration of macrophages and neutrophils, black arrows represent multifocal necrosis and degeneration, and green arrows represent viral RNA in neurons and at sites of inflammation.

Supplementary Figure S3. Survival, weight change and clinical score following challenge with ma-CHAPV in STAT-1$^{-/-}$ mice. Six- to ten-week-old STAT-1 knockout mice were infected with 1000 pfu of ma-CHAPV via the intraperitoneal route and monitored daily for (A) survival, (B) weight change and (C) clinical score for 48 days post-challenge. Data is representative of one experiment of *n* = 20 mice.

Supplementary Figure S4. Weight change and clinical score following challenge with ma-CHAPV in STAT1$^{-/-}$ mice. Four- to twelve-week-old STAT1 knockout mice were infected with 1000 pfu of ma-CHAPV via the intraperitoneal route and monitored daily for (A) weight change and (B) clinical score for 36 days post-challenge. Error bars represent SEM. Data is representative of one study of *n* = 32 mice with *n* = 4 mice harvested days 4, 8, 12, 18, 20, 23, 29, and 36 post-challenge. Mice were separated into two pans of females (*n* = 8, 10 per pan) and two pans of males (*n* = 10, 4 per pan), group weighed, and pan weights reported.
