# Supplementary figures and images for: A STAT1-Knockout Mouse Model for Chapare Virus Infection and Pathogenesis
Source: Viruses. 2026 Mar 20;18(3):388. doi: 10.3390/v18030388 (PMC13030867; doi:10.3390/v18030388)

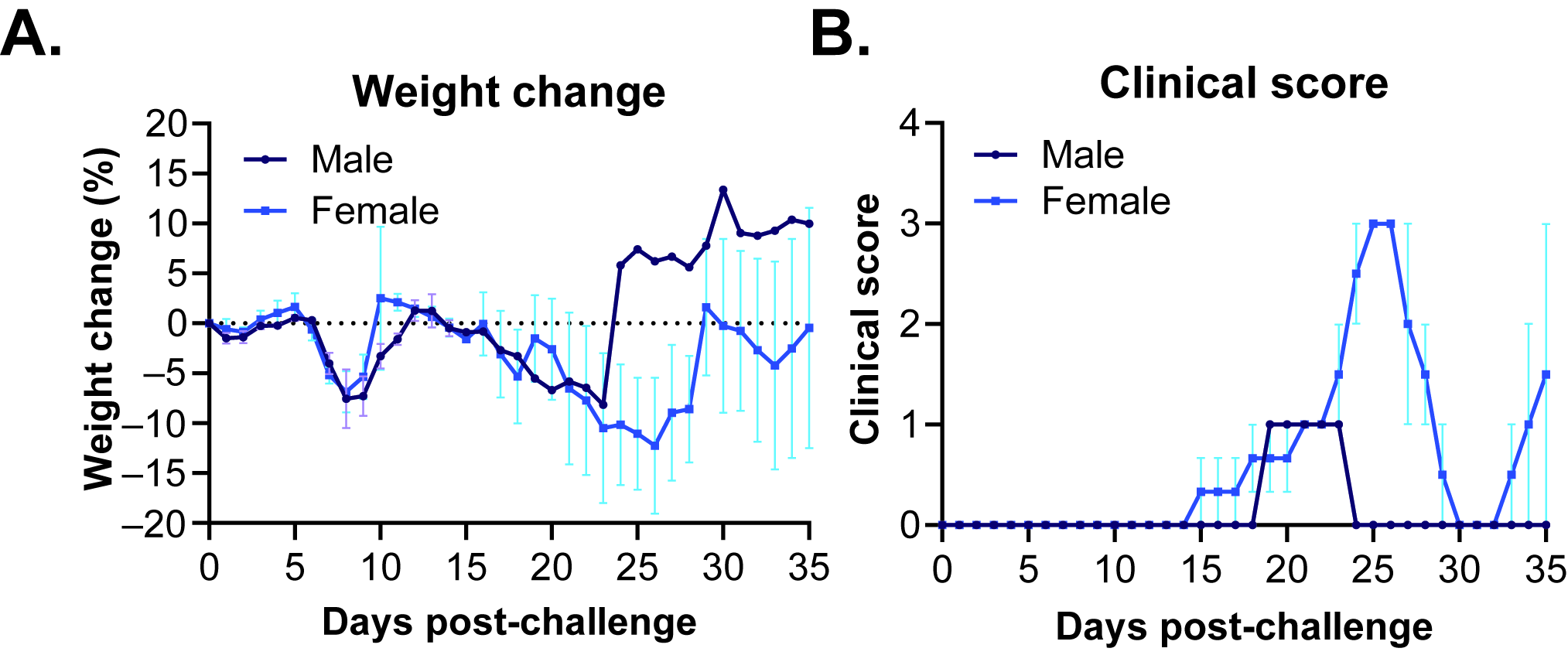

Supplement: Supplementary file 1 [file viruses-18-00388-s001.zip › Supplementary Figure S1.tif]

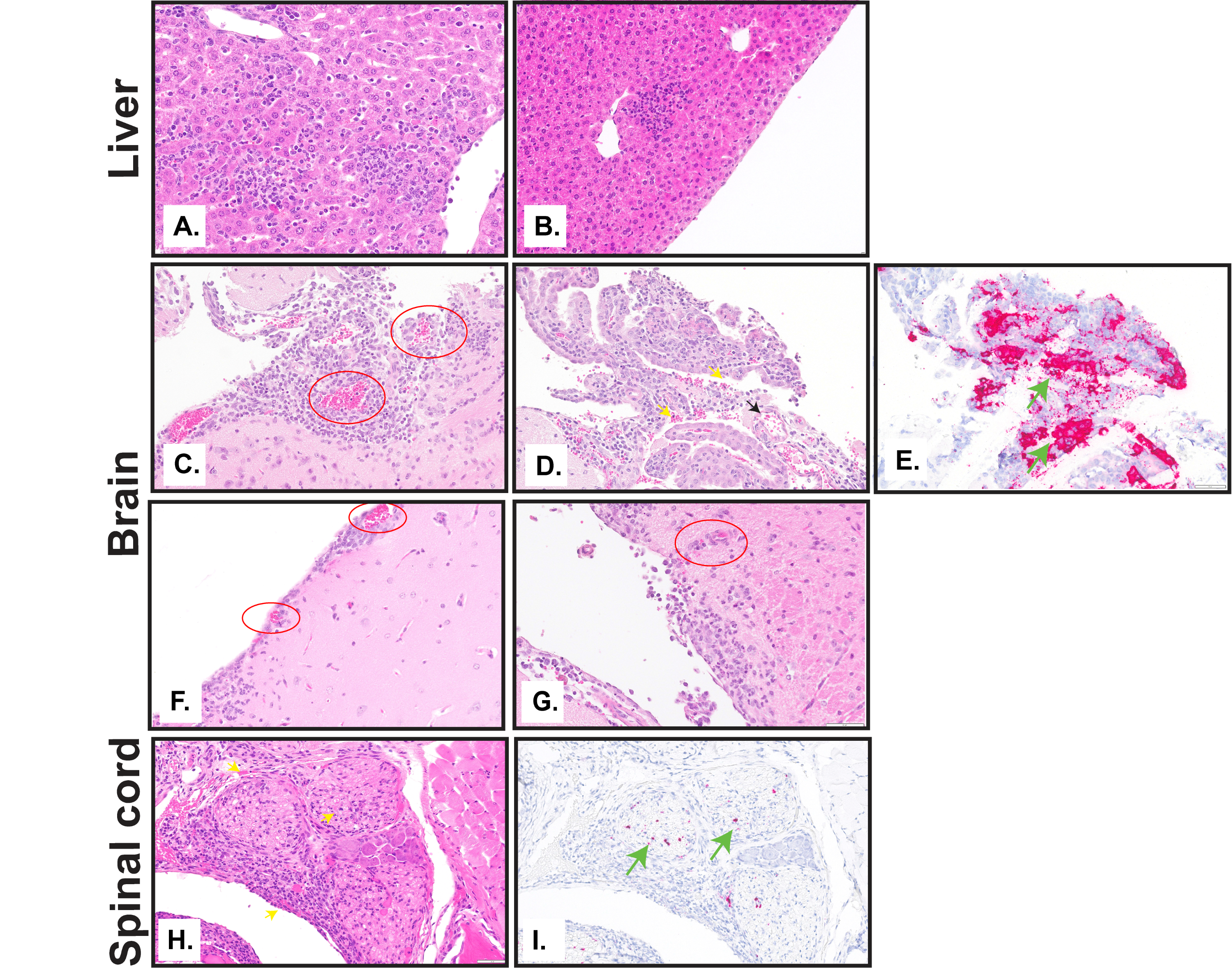

Supplement: Supplementary file 1 [file viruses-18-00388-s001.zip › Supplementary Figure S2 v2.tif]

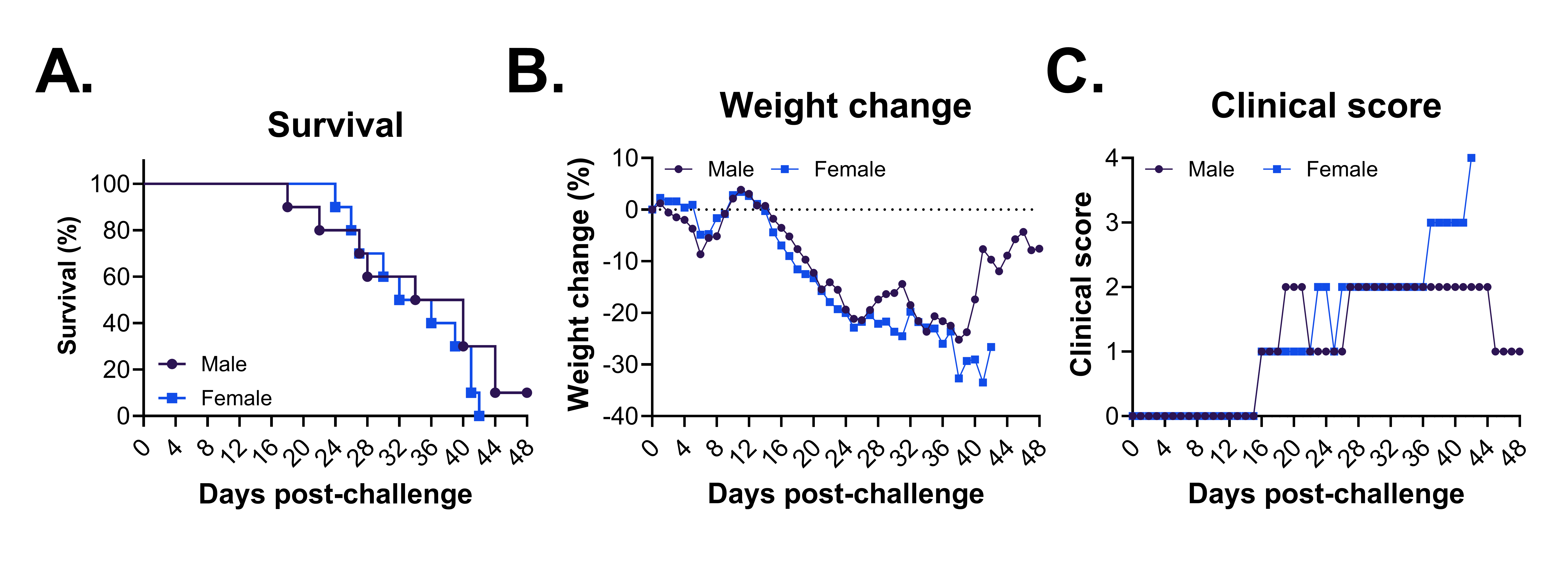

Supplement: Supplementary file 1 [file viruses-18-00388-s001.zip › Supplementary Figure S3.tif]

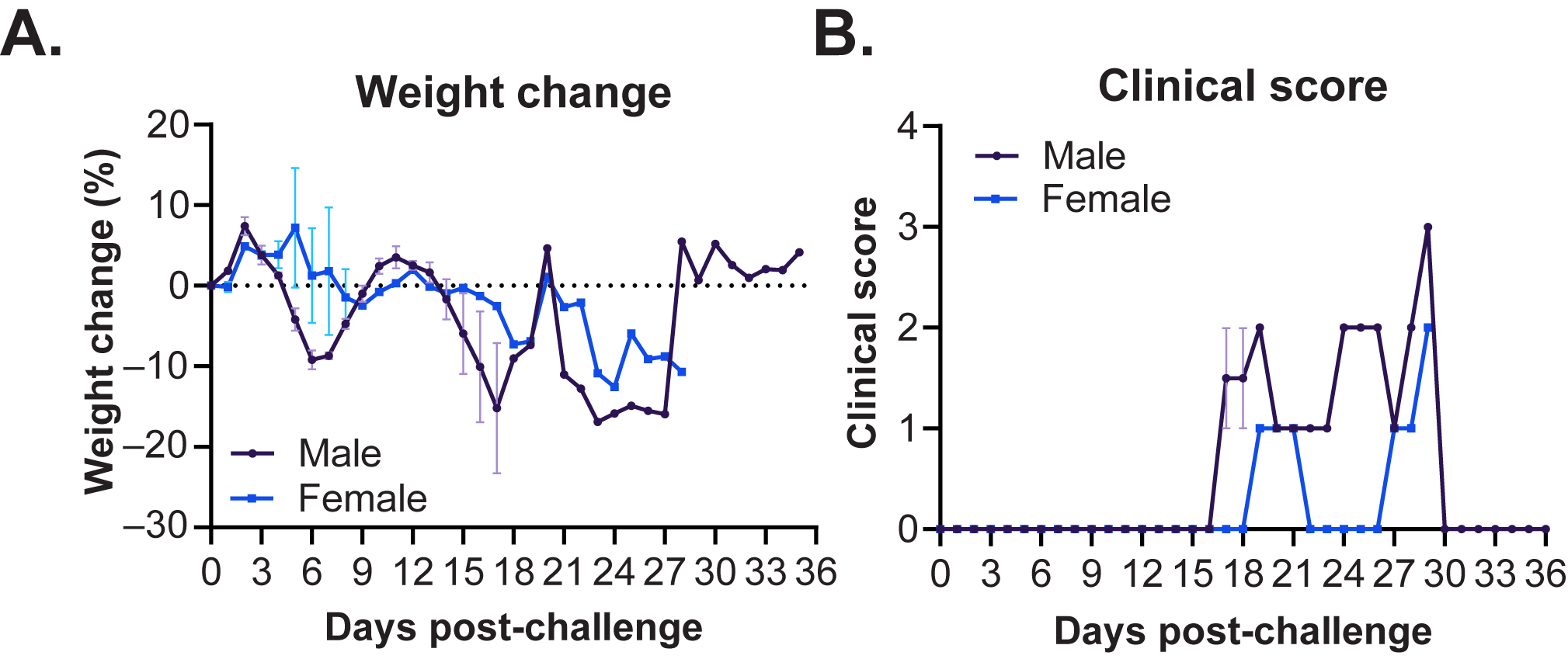

Supplement: Supplementary file 1 [file viruses-18-00388-s001.zip › Supplementary Figure S4.tif]
